# A case of ‘fat-free’ pleomorphic lipoma occurring in the upper back and axilla simultaneously

**DOI:** 10.1186/1477-7819-11-145

**Published:** 2013-06-20

**Authors:** Liang Wang, Yang Liu, Di Zhang, Yong Zhang, Na Tang, En-Hua Wang

**Affiliations:** 1 Department of Pathology, the First Affiliated Hospital and College of Basic Medical Sciences, China Medical University, North 2nd Road, 92#, Shenyang 110001, China; 2 Institute of pathology and pathophysiology, China Medical University, North 2nd Road, 92#, Shenyang 110001, China

**Keywords:** Spindle cell lipoma, Pleomorphic lipoma, Atypical lipomatous tumor, Myxoid liposarcoma, Pleomorphic hyalinizing angiectatic tumor

## Abstract

Pleomorphic lipoma is a rare neoplasm that predominantly occurs in the dermis or subcutis of the posterior neck, upper back, and shoulders. Although pleomorphic lipoma is a benign tumor, it may contain atypical cells. As a variant of spindle cell lipoma, pleomorphic lipoma clinically presents as a slow-growing and well-circumscribed subcutaneous mass. Rarely, some patients have multiple lesions. Histologically, pleomorphic lipoma is composed of mature fat, bland spindle-shaped mesenchymal cells, and coarse ‘rope-like’ collagen bands. In addition, lipoma contains multinucleated floret-like giant cells. Although spindle cell lipoma/pleomorphic lipoma with little fat was seen in the original series described by Enzinger and Harvey, cases with little to no fat remain diagnostically challenging. Herein, we report a case of ‘fat-free’ pleomorphic lipoma occurring in the upper back and axilla simultaneously. Although the lipoma was typically composed of bland spindle-shaped cells, rope-like collagen, scattered floret-like giant cells, and striking stromal myxoid change in the background, mature fat was absent. Immunohistochemical analyses showed positive staining for CD34, vimentin, and Bcl-2, and negative staining for S100, confirming the diagnosis of pleomorphic lipoma.

## Background

Pleomorphic lipoma is a rare, benign, pseudosarcomatous, soft-tissue neoplasm that typically involves the subcutis of the posterior neck, upper back, and shoulders. Pleomorphic lipoma is a variant of spindle cell lipoma, and these two types of lipoma exhibit similar histological features and immunophenotypes. A recent study demonstrated that spindle cell lipoma and pleomorphic lipoma have the same genetic aberrations, such as partial loss of chromosome 13 and/or 16, and mostly deletions of 16q13-qter [1].

Both pleomorphic and spindle cell lipomas typically present in older men (85 to 90%), with a median age of >55 years. The patients often have a long history of these lesions. Although most pleomorphic and spindle cell lipomas occur in the aforementioned locations, they can occasionally involve the palm, tonsillar fossa, orbit, tongue, vulva, and oral cavity [2-7]. However, it is still debated whether lesions occurring in these rare locations should be diagnosed as spindle cell lipoma/pleomorphic lipoma instead of atypical lipomatous tumor [8,9]. Pleomorphic and spindle cell lipomas are usually relatively small (2 to 5 cm), but can occasionally reach sizes >10 cm. Some patients have multiple lesions, and familial occurrence has also been reported, but mostly in men [1].

Histologically, pleomorphic lipoma consists of mature fat, bland spindle-shaped mesenchymal cells, ‘rope-like’ collagen bands, and ‘floret-like’ cells. Floret-like cells are characterized by their radially arranged nuclei, resembling the petals of flowers. Some pleomorphic lipomas have prominent nuclear atypia with hyperchromatism and even occasional atypical mitoses. In such instances, the border between pleomorphic lipoma and atypical lipomatous tumor is blurred. In addition, the amount of mature fat in pleomorphic lipoma is variable [10,11], which poses a great diagnostic challenge in cases with little to no fat. Immunohistochemically, pleomorphic lipoma is strongly positive for CD34, but negative for S100 protein and smooth muscle actin (SMA) [1].

Since pleomorphic lipoma resemble a sarcoma, histopathologic diagnosis is critical for preventing unnecessary surgery. Here, we report a case of pleomorphic lipoma with no fat that occurred in a 65-year-old Chinese male patient.

## Case presentation

### Clinical history

A 65-year-old male was admitted to the First Affiliated Hospital of China Medical University in June 2011 for further examination of masses occurring in the upper back and right axilla that were found accidentally. A physical examination revealed 3-cm and 6-cm subcutaneous masses in the upper back and right axilla, respectively. Ultrasonography revealed two well circumscribed and low-echo masses in the subcutaneous fat tissue of the aforementioned locations. The tumors were excised and biopsies were taken. The patient was still alive with no tumor recurrence or metastasis after 17 months of follow-up.

### Gross features

Gross examination showed two masses with the diameter of 3 cm (upper back) and 6 cm (right axilla). The tumors were grayish-white on their cut surfaces, had a firm texture, and were clearly demarcated from the adjacent normal tissues.

### Microscopic features

**Figure 1:**
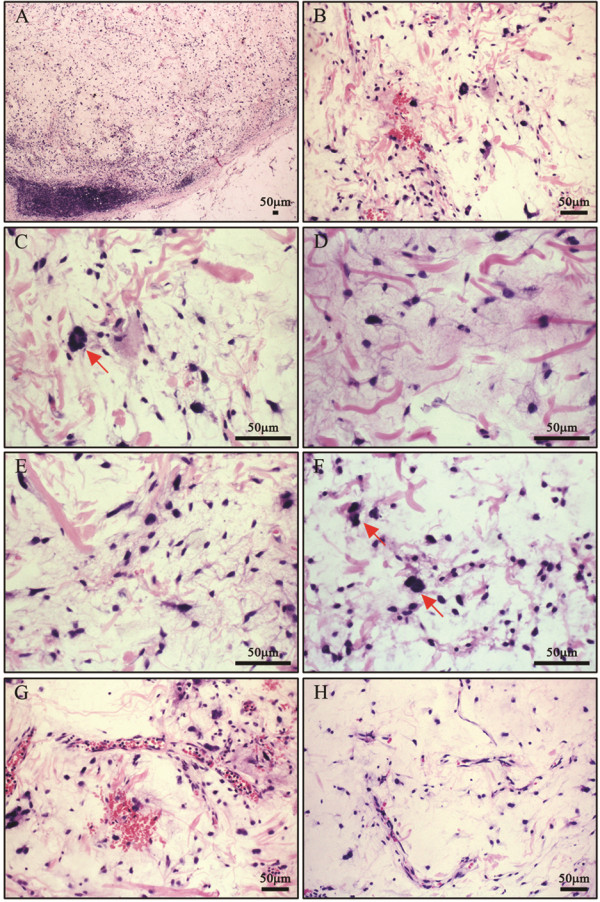
**Histological features. A**) The tumor was demarcated from the surrounding tissues with a relatively clear boundary (40×). **B**) The tumor showed striking floret-like giant cells and stromal myxoid change in the background (200×). **C**) The floret-like giant cells (arrow) had radially arranged nuclei and showed striking nuclear atypia (400×). **D**) Irregular rope-like collagen was arranged in the diffuse myxoid stroma (400×). **E**) Hypocellular bland spindle-shaped cells were uniform with hyperchromatic nuclei and inconspicuous nucleoli (400×). **F**) Atypical cells (arrow) were scattered in the background (400×). **G**) A few small or intermediate-sized, thick-walled vessels were observed (200×). **H**) Focally, the plexiform vascular pattern was also observed in this case (200×).

The tumors in the two different locations had similar histological features. The tumors were demarcated from the surrounding tissues with a relative clear boundary, and presented with striking floret-like giant cells and stromal myxoid change in the background. In addition, hypocellular bland spindle-shaped cells admixed with irregular rope-like collagen were arranged in a diffuse myxoid stroma. The spindle-shaped cells were uniform with hyperchromatic nuclei and inconspicuous nucleoli. The floret-like giant cells had radially arranged nuclei and showed striking nuclear atypia. However, mitoses and mature fat were rarely seen. A few small or intermediate-sized, thick-walled vessels were observed. Focally, a plexiform vascular pattern, which resembled that in myxoid liposarcoma, was also observed in the present case (Figure 1).

### Immunohistochemistry

**Figure 2:**
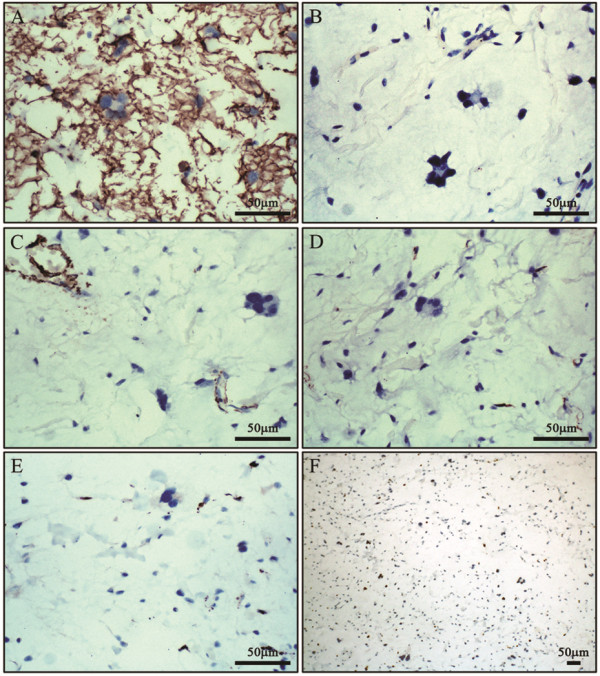
**Immunohistochemical staining. A**) Diffuse and strong CD34 staining highlighted the tumor cells (400×). **B**) The spindle cells and floret-like cells were negative for S100 (400×). **C**) The spindle cells and floret-like cells were negative for SMA, while SMA staining highlighted the dilated vessels (400×). **D**) The spindle-shaped cells and floret-like cells were negative for desmin (400×). **E**) The spindle-shaped cells and floret-like cells were negative for CD68 (400×). **F**) The Ki67 index was less than 1% (100×).

Immunohistochemical analyses showed that the bland spindle-shaped mesenchymal cells and floret-like cells were positive for CD34, vimentin, and Bcl-2, but negative for S100, desmin, SMA, and CD68. The Ki67 index was about 1% (Figure 2).

## Discussion

Pleomorphic lipoma is a relatively uncommon benign lipomatous tumor that shows a variable, nonlipogenic, spindle-shaped cell component and floret-like giant cells with nuclear pleomorphism, and was first described by Shmookler and Enzinger in 1981 [10]. It is also the histologic variant of spindle cell lipoma described by Enzinger and Harvey in 1975 [12]. Pleomorphic lipoma typically appears in the subcutis of the head or neck as a slowly growing mass. The average time between appearance and diagnosis is about 3 years. Pleomorphic lipoma is four times more common in males and usually presents between the ages of 50 and 70 years. A histologic diagnosis is best made on formalin-fixed and paraffin-embedded sections of dissected specimens, because the pleomorphic cells can masquerade as malignant cells on fine-needle aspiration biopsy.

Although pleomorphic lipoma mimics various soft-tissue tumors, it can be recognized or suspected on morphologic grounds. The clues to the diagnosis are: 1) predominantly located in the subcutis or dermis of the posterior neck, upper back, and shoulders; 2) variable amount of adult fat, rarely fat-free and no lipoblasts; 3) atypical cells ranging from rare to frequent; 4) bundles of dense rope-like collagen; 5) possible myxoid stroma; and 6) CD34-positivity, usually extensive.

The histologic differential diagnosis of classic pleomorphic lipoma includes both benign and malignant soft tissue neoplasms, such as fat necrosis, pleomorphic hyalinizing angiectatic tumor (PHAT), spindle cell lipoma, atypical lipomatous tumor and myxoid liposarcoma. Pleomorphic lipoma may be confused with fat necrosis because of the nodular appearance. In fat necrosis, foamy cells frequently fill the spaces left by dead adipocytes and rope-like collagen is absent. These features will be helpful for the diagnosis. Pleomorphic lipoma should also be distinguished with PHAT. Intracytoplasmic hemosiderin and aggregates of vessels with hyalinized walls and fibrin are always seen in PHAT, but are absent in pleomorphic lipoma. In addition, PHAT seldom occurs in the posterior neck, upper back, or shoulders, and generally contains no rope-like collagens. As mentioned earlier, spindle cell lipoma and pleomorphic lipoma are part of a spectrum. They display overlapping histological features and similar immunophenotypes. Atypical lipomatous tumor is the most difficult one among the differential diagnosis of pleomorphic lipoma. The histological features and atypical cells observed in atypical lipomatous tumor make it nearly impossible to distinguish from pleomorphic lipoma on morphologic grounds. The floret-like giant cells in pleomorphic lipoma can occasionally also be seen in atypical lipomatous tumor. Thus, the absence of floret-like cells cannot distinguish the two lesions. In such cases, locations of the lesions can provide some clues to make the final diagnosis. Pleomorphic lipoma usually arises in the subcutaneous tissue of the posterior neck, upper back, and shoulders, while atypical lipomatous tumor usually arises in deep soft tissue of the extremities. In addition, pleomorphic lipoma should also be distinguished from myxoid liposarcoma. The typical microscopic appearance of myxoid liposarcoma includes sheet-like areas of proliferating lipoblasts in varying stages of differentiation, a plexiform capillary pattern mostly at the edge of the tumor lobules, and a myxoid matrix between the vessels and tumor cells. The lipoblasts have a signet-ring appearance, and scattered spindle-shaped cells, multinucleated lipoblasts, and giant cells may also be present. Occasionally, myxoid liposarcoma may have areas with increased cellularity and exhibit extreme nuclear pleomorphism, and typically contains giant lipoblasts with bizarre, hyperchromatic, and scalloped nuclei. However, in such cases, many atypical mitotic figures, necrosis, and hemorrhage are always present, together with giant cells. Moreover, pleomorphic lipoma lacks lipoblasts, which can be seen in myxoid liposarcoma [13,14]. In addition, CD34 and S100 will be helpful for the diagnosis.

Very rarely, pleomorphic lipoma may entirely lack a lipomatous component, and pose a great diagnostic challenge. Thus far, there is only one report in the literature describing two cases of pleomorphic lipoma with devoid of mature fat tissue. This tumor was designated the fat-free variant of pleomorphic lipoma [15]. In our case, spindle cells and floret-like cells were scattered in an extensive myxoid stroma, and the adipose tissue was absolutely absent. Owing to the absence of fat, we first thought that it might be a myxoid fibrosarcoma. Myxoid fibrosarcoma is more common than pleomorphic lipoma in elder males. The majority of these tumors occur in the extremities, and rarely on the trunk or head and neck area. Histologically, myxoid fibrosarcoma usually consists of spindle-shaped cells and multinucleate giant cells, and is characterized by prominent elongated, curvilinear, thin-walled blood vessels. Although the spectrum of myxoid fibrosarcoma is also variable, it tends to have more cellular atypia and mitotic activity. Moreover, myxoid fibrosarcoma usually lacks strong CD34 expression [16]. In addition, pleomorphic lipoma should be distinguished with giant cell fibroblastoma, which is also positive for CD34 expression and possesses multinucleate giant cells. However, giant cell fibroblastoma is a juvenile form of dermatofibrosarcoma protuberans, and predominantly affects infants and children. It is characterized by multinucleate tumor cells lining slit-like pseudovascular spaces [17]. On the contrary, pleomorphic lipoma mainly occurs in elder men.

In our case, the patient had multiple lesions on the upper back and axilla, with well-demarcated surroundings. Although the histopathological features suggested the diagnosis of pleomorphic lipoma, it remained challenging because of the lack of a fat component. Recently, Sachdeva [11] and Billings [18] reported the ‘low-fat’ and ‘fat-free’ variants of pleomorphic lipoma and spindle cell lipoma, respectively. Meanwhile, they proposed that the key to the diagnosis of such variants lies in the nonlipogenic component rather than the lipogenic component. In the present case, the histopathological features and immunophenotypes of the floret-like giant cells and spindle-shaped cells coincided with those of typical pleomorphic lipoma. For these reasons, the final diagnosis was pleomorphic lipoma.

## Conclusions

In conclusion, pleomorphic lipoma is a rare benign soft-tissue neoplasm that can resemble a variety of malignant soft-tissue tumors. Therefore, careful examination of the histopathological characteristics and immunophenotypes is essential for reaching a correct diagnosis, thereby avoiding unnecessary disfiguring surgery.

## Consent

Written informed consent was obtained from the patient for publication of this case report and accompanying images. A copy of the written consent is available for review by the Editor-in Chief of this Journal.

## Competing interests

The authors declare that they have no competing interests.

## Authors’ contributions

LW analyzed the data and wrote the manuscript as a major contributor. DZ and NT helped to perform the immunochemical staining. YL, YZ and EW helped to revise the discussion section of this manuscript. All authors have read and approved the final manuscript.
